# Comparative Economic Evaluation of Radical Prostatectomy, Radiation, and Ablative Techniques in the Management of Localized Prostate Cancer

**DOI:** 10.3390/cancers17172814

**Published:** 2025-08-28

**Authors:** Mahdi Mottaghi, Alireza Ghoreifi, Sriram Deivasigamani, Eric S. Adams, Sudharshanan Balaji, Michael C. Ivey, Cary N. Robertson, Judd W. Moul, Ryan E. Fecteau, Thomas J. Polascik

**Affiliations:** 1 Department of Urology, Duke University Medical Centre, Durham, NC 27710, USA; 2 Durham Veteran Affairs Health System, Institute for Medical Research, Durham, NC 27710, USA; 3 Department of Radiology, Duke University Medical Center, Durham, NC 27710, USA; 4 Duke Cancer Institute, Duke University Medical Centre, Durham, NC 27710, USA; ryan.fecteau@duke.edu

**Keywords:** cost, cryotherapy, high intensity focused ultrasound ablation, implant radiotherapy, prostate cancer, prostatectomy, radiation therapy

## Abstract

**Simple Summary:**

Men with low- to intermediate-risk prostate cancer have several treatment options, from active monitoring to surgery or radiation. This research looked at the actual expenses of seven common treatments to help patients, doctors, and healthcare decision-makers better understand the financial implications. We found that treatments requiring more sessions or advanced technology generally cost more. Please refer to the graphical abstract for more information.

**Abstract:**

**Background:** To compare the costs of open retropubic radical prostatectomy (RRP), robotic-assisted radical prostatectomy (RALP), intensity-modulated radiation therapy (IMRT), low-dose brachytherapy (LDBT), stereotactic body radiotherapy (SBRT), cryotherapy (Cryo), and high-intensity focused ultrasound (HIFU) for low/intermediate-risk prostate cancer (PCa), from the healthcare system perspective. **Methods:** This retrospective, IRB-approved study compared the costs and charges of primary treatment options for localized PCa at Duke University Hospital between January 2018 and December 2019. We identified cases by querying the relevant disease, procedural, and charge codes from Duke Finance. Consecutive cases with NCCN high-risk disease, prior treatment, or missing institutional financial information were excluded. Costs were calculated from the point at which the treatment option was selected until the last treatment session (SBRT and IMRT) or hospital discharge (other modalities). All modalities except RRP were considered technology-intensive. **Results:** A total of 552 patients with a mean age of 65.0 years met the inclusion criteria. NCCN risk categories included 85 (13%) low, 218 (41%) favorable-intermediate, and 249 (46%) unfavorable-intermediate risk cases. RALP, RRP, Cryo, and HIFU were single-session treatments, whereas IMRT, SBRT, and LDBT were delivered over multiple sessions. IMRT and SBRT were the most expensive modalities, followed by RALP, HIFU, LDBT, Cryo, and RRP. The number of sessions (ρ = 0.55, *p* < 0.001) and being technology-intensive (ρ = 0.58, *p* < 0.001) were significantly correlated with treatment costs. **Conclusions:** In this cohort of PCa patients, treatment costs were highest for IMRT and SBRT, followed by RALP, HIFU, LDBT, Cryo, and RRP. The number of treatment sessions was a significant predictor of higher costs.

## 1. Introduction

The current management of localized, low- to intermediate-risk prostate cancer (PCa) includes a range of approaches, ranging from active surveillance to definitive treatments such as radical prostatectomy and radiation therapy [1,2]. Additionally, minimally invasive ablative therapies could be considered in select patients, particularly within clinical trials or prospective registries, with the primary aim of preserving quality of life [2,3,4]. The current guidelines recommend a multidisciplinary approach before deciding on the management of localized PCa due to the wide range of available options and their potential side effects [5,6,7]. In addition, the cost of treatment is a critical factor in the decision-making process for patients, clinical specialists, and policymakers. It has been shown that over half of cancer patients are willing to discuss the economic aspects of their treatment; however, less than a third of them receive sufficient counseling on these financial issues [8].

Although clinical and patient-centered outcomes of the aforementioned treatment options have been extensively studied, direct comparisons of treatment costs remain relatively scarce, particularly between conventional options and minimally invasive modalities such as cryotherapy (Cryo), high-intensity focused ultrasound (HIFU), and low-dose brachytherapy (LDBT) [9]. A British cost-effectiveness study compared focal Cryo and focal HIFU to robotic-assisted radical prostatectomy (RALP) and external-beam radiation therapy (EBRT), reporting higher quality-adjusted life year gains for the focal options [10]. Furthermore, a recent German cost-utility analysis compared MRI-guided transurethral ultrasound ablation (TULSA) with active surveillance (AS), RALP, and EBRT, and showed that for a low- to intermediate-risk PCa and considering a willingness-to-pay of approximately €14,000, the probability of being cost effective is similar between AS, RALP, and TULSA, while higher thresholds of willingness-to-pay favored AS [11]. Given the lack of contemporary economic evaluations in the U.S. health setting, we aimed to conduct a cost comparison of open retropubic radical prostatectomy (RRP), RALP, intensity-modulated radiation therapy (IMRT), LDBT, stereotactic body radiotherapy (SBRT), Cryo, and HIFU for low- to intermediate-risk PCa at a tertiary referral center, from the healthcare system perspective.

## 2. Materials and Methods

### 2.1. Patient Population

We conducted a retrospective cohort study at Duke University Hospital to compare institutional costs and charges associated with various primary treatment modalities for eligible consecutive patients with localized PCa treated between 1 January 2018 and 31 December 2019. The study received approval from the institutional review board. Patients were identified by querying financial records for their medical record numbers associated with International Classification of Diseases, Tenth Revision, Clinical Modification (ICD-10-CM) code “C61” who had at least one registered encounter during the specified study period. These cases were further narrowed using procedure-specific Current Procedural Terminology (CPT) codes (Supplementary Material, Table S1) for surgical procedures (RRP, RALP, Cryo, HIFU), financial charge codes maintained by Duke Finance for radiation modalities (IMRT, SBRT, LDBT), and a manual review to ensure capture of all eligible cases. To maintain data homogeneity, cases were excluded if patients had high-risk disease according to National Comprehensive Cancer Network (NCCN) guidelines, had received prior or repeat PCa treatment, or had incomplete financial data on file. Of note, the selection of treatment modalities involved a shared decision-making process between patients and physicians.

### 2.2. Study Variables and Definitions

Study variables included patient demographics (age, race), NCCN risk groups, treatment modality, number of treatment sessions, costs, institutional charges, and use of modality-specific procedures such as fiducial placement and hydrogel spacer injection for IMRT and SBRT, prostate volume study, simulation, dosimetry, and seed placement for LDBT, focal versus whole-gland pattern of treatment for ablation modalities. The primary objective of the study was to report and compare the mean and standard deviation (SD) of total and direct institutional costs and total charges. The secondary objective was to assess the correlation between treatment characteristics (e.g., session number, technology-intensiveness) and costs. The exploratory objectives were to report the costs and charges of spacer gel injection for IMRT and SBRT, as well as the costs and charges of post-treatment biopsy and mpMRI for ablation modalities.

In this study, “cost” denotes the actual expense incurred by a provider to deliver a service, whereas “charge” refers to the price assigned by the provider for that service. Direct costs are those associated with patient care, such as procedures, medications, and clinical staff, while indirect costs are necessary but non-clinical expenses, such as administrative support, facility operations and maintenance, utilities, cleaning and sanitation services, bedding, uniforms, and protective equipment. The overall costs (direct plus indirect costs) were captured from the time a treatment modality was selected through to the completion of that treatment. This study has not captured costs related to the treatment complications and androgen deprivation therapy for any of the mentioned treatment options. For radiation therapy modalities (SBRT, IMRT, and LDBT), this period extended to the final radiation session, while for RALP, RRP, HIFU, and Cryo treatments, cost analysis was conducted through hospital discharge. Diagnostic services, such as multiparametric MRI, diagnostic prostate biopsy, or potential genomic evaluations, were excluded from cost calculations if performed prior to the assignment of the treatment plan. For radiation therapy, additional procedure-specific expenses related to fiducial marker placement, hydrogel spacer injection, and dosimetry were also included (Table 1). Regarding ablative modalities, the costs and charges of focal and whole-gland techniques were kept unified in the cost comparison analysis due to the similar CPT code. All treatments except RRP were categorized as technology-intensive due to their reliance on specialized equipment and planning protocols. The discount rate was not applied, given the 2-year study period.

### 2.3. Statistical Analysis

Continuous variables, such as direct and total costs, were summarized using means and SDs, while categorical variables, including treatment type and risk classification, were described as frequencies and percentages. Economic evaluations mostly use mean and standard deviation. However, due to the sample size considerations, we reported both the mean and the median for better illustration of the data spread. The Chi-squared test was used to compare the race and risk groups across treatment modalities. To compare age differences across treatment groups, we used a One-way ANOVA test with Tukey post hoc comparisons. The Mann–Whitney U test with Bonferroni correction was used for pair-wise cost comparisons. Relationships between treatment costs and factors such as the number of treatment sessions or use of technology-intensive modalities were evaluated using Spearman’s correlation. All statistical analyses were performed using SPSS version 26 (IBM Corp., Armonk, NY, USA, https://www.ibm.com/products/spss, accessed on 8 September 2024), with statistical significance set at a *p*-value of <0.05, unless corrected with Bonferroni, which is mentioned in the results when applicable.

## 3. Results

We identified 633 cases of low- to intermediate-risk PCa patients who underwent primary treatment at our center. Of these, 83 cases were excluded due to missing financial data. Of note, 73 of 83 excluded patients belonged to the IMRT cohort, mostly because they received at least one part of the treatment (fiducial placement or some radiation sessions) outside our center.

A total of 550 patients met the inclusion criteria during the study period. Of these, 372 (68%) were White, 149 (27%) patients were Black, 14 (2%) belonged to other ethnic backgrounds, and 15 (3%) did not declare their race. Table 2 presents details on age, race, NCCN risk groups, and the number of sessions for each modality. The mean (SD) age of all participants was 65.0 ± 7.2 years. Pairwise Mann–Whitney comparisons with Bonferroni corrections showed that participants in Cryo (69.43 years) and IMRT groups (69.27 years) were statistically significantly older (*p* < 0.0024) than those in the LDBT (64.54 years), RALP (62.91 years), and RRP groups (62.83 years). NCCN low-, favorable-intermediate-, and unfavorable-intermediate-risk categories were identified in 85 (13%), 218 (41%), and 249 (46%) patients, respectively. RALP, RRP, Cryo, and HIFU were single-session interventions, whereas IMRT, SBRT, and LDBT were delivered over multiple sessions. The number of radiation delivery sessions is provided in Table 2. Of note, an additional 2–3 visits for fiducial placement, simulation, and dosimetry should also be considered for IMRT and SBRT.

Table 3 outlines the breakdown of mean total costs, direct costs, and overall institutional charges by treatment type. IMRT and SBRT were the two most resource-intensive modalities, with mean total costs of USD 28.5 K (±6.0 K) and USD 20.3 K (±1.7 K), and charges averaging USD 136.2 K (±10.0 K) and USD 121.3 K (±6.9 K), respectively. RALP was the third most costly modality, with mean total costs of USD 14.0 K (±3.3 K) and overall charges averaging USD 40.3 K (±6.6 K). On the other hand, RRP was the least costly treatment overall, followed by Cryo, LDBT, and HIFU. Among focal therapy modalities, Cryo had an average total cost of USD 10.5 K (±1.4 K), and HIFU showed a mean total cost of USD 12.9 K (±1.5 K).

Supplementary Tables S2–S4 present the detailed costs for LDBT, IMRT, and SBRT modalities, respectively. Regarding LDBT, seed placement was the most expensive step, followed by the prostate volume study, simulation, and post-implant dosimetry. We could not separate the IMRT and SBRT costs similar to LDBT, as several patients received simulation and fiducial placement in a single session.

Supplementary Tables S3a and S4 break down the costs and charges for patients who received hydrogel spacer injections compared to those who did not. As anticipated, spacer injections incurred significantly higher charges; nevertheless, higher total and direct costs were observed for patients without spacer injections for both SBRT and IMRT cohorts. This unexpected finding prompted further investigation. As shown in Table S3b, the location of service caused the unexpected difference in costs; all spacer gel injections were delivered in the interventional radiology clinic, in a community hospital, while the fiducial placements without a spacer were performed in the academic center, either in the clinic or the ambulatory surgery facility’s operative room. Table S5 provides costs and charges for Cryo and HIFU modalities, stratified by ablation pattern used (focal versus whole-gland). The Mann–Whitney test did not show a significant difference between the focal and whole-gland subgroups for each modality.

Statistical analysis revealed a significant positive correlation between the number of treatment sessions and total cost (Spearman’s ρ = 0.55, *p* < 0.001), indicating that multi-session therapies tend to incur higher expenses. Similarly, classification as a technology-intensive modality was also significantly correlated with overall cost (ρ = 0.58, *p* < 0.001).

Among the ablation cohort (68 cases overall), 38 patients underwent follow-up multi-parametric MRI, and 17 underwent biopsy. The costs and charges for these cases were not included in the cost comparison analysis per our inclusion criteria but are reported separately as complementary information (Table S6). Surveillance MRI and biopsy added USD 0.9 K (±0.2 K) and USD 4.0 K (±1.0 K) to the costs and USD 6.5 K (±1.7 K) and USD 9.0 K (±4.5 K) to the charges, respectively.

## 4. Discussion

To our knowledge, this is the first cost-comparison study to report on seven treatment modalities for PCa. We identified significant variation in associated costs across treatment modalities. While the majority of patients were White and classified within intermediate-risk NCCN categories, notable differences in age distribution were observed, with older patients more likely to receive Cryo or IMRT. Treatment modality played a key role in cost differentiation, with IMRT and SBRT being the most expensive options, both in total costs and institutional charges, followed by RALP, HIFU, LDBT, Cryo, and RRP. Multi-session and technology-intensive interventions were strongly correlated with higher costs.

Extracting and evaluating IMRT charges are challenging and vary significantly between institutions; Agrawal et al. evaluated 52 hospitals of National Cancer Institute-designated cancer centers and showed IMRT “delivery-only” charges could vary by a 21.7-fold difference between the least and most expensive hospitals [12].

Among radiation modalities, the higher number of sessions in the IMRT cohort resulted in higher costs compared to SBRT. When interpreted in the context of the PACE-B trial, which demonstrated comparable oncological efficacy and toxicity profiles between the two radiation modalities, SBRT appears to be a more cost-effective option [13,14]. However, IMRT and SBRT were more than twice as expensive as LDBT, which is comparable to the findings from the national Medicare data analysis conducted by Tang and colleagues [15]. Regarding both IMRT and SBRT, a surprising finding was that although the spacer injections led to significantly higher charges, the costs were unexpectedly lower for these patients. Further investigation showed that this difference was due to the location where the services were performed, as all spacer gel injections occurred at an interventional radiology clinic in a community hospital. In contrast, fiducial placements without a spacer were performed at the academic center (in either a clinic or an ambulatory surgery facility) with a higher cost structure in general and a different revenue model, compared to the community hospital.

Our study found that RALP costs were almost 1.5 times higher, and its charges were nearly twice that of RRP. Although post-operative costs were not included in our analysis, previous research has shown that the higher upfront costs of RALP could be offset within the first year due to increased healthcare use following open RP [16]. Notably, a recent meta-analysis by Bejrananda et al., evaluating the economic outcomes of RALP versus open RP in localized PCa, revealed that both techniques are comparable in terms of cost-effectiveness. However, RALP appears to offer a more favorable long-term economic profile, while open RP remains a financially viable option in both high- and middle-income countries, particularly in the latter [17]. Another interesting finding was that the proportion of Black men was lower in the RRP cohort compared to the IMRT group. While it has been shown that Black men with low-intermediate risk PCa are more likely to undergo radiation therapy compared to RP [18], the costs are not expected to be different based solely on race, and the reasons for the higher tendency toward a specific treatment option based on race should be investigated in the future.

Focal ablative therapies for intermediate-risk PCa are relatively new alternatives for the definitive management of PCa, with promising trials showing favorable mid- and long-term oncological and functional outcomes [2,4,19,20]. The American Urological Association and European Association of Urology guidelines recommend offering focal ablative therapies to highly selected patients within clinical trials or prospective registries [2,4]. A meta-analysis in 2023 on the primary whole-gland Cryo and HIFU ablation included 29 studies with a minimum sample size of 50 patients, showing that cancer-specific, overall, and metastasis-free survival rates at 10 years were 96%, 63%, and 84%, respectively [21]. Multiple multidisciplinary consensus statements recommended post-ablation imaging for all focal therapy patients, and biopsy was mostly recommended to be performed “for cause” rather than “per protocol” [22]. The MRI cost was not included in the main cost comparison for ablation modalities because our cohort included both focal and whole-gland ablations, and not all patients undergo MRI; however, a separate comparison accounting for MRI cost was provided in the Supplementary Material. The costs and charges of whole-gland treatment did not statistically differ from those of focal ablation. Biopsy costs were also not included in the comparison. The rationale was that a for-cause biopsy, similar to the workup for biochemical recurrence after definitive treatment, necessitates additional evaluation, such as biopsy following ablation and further imaging after definitive management.

Conducting economic evaluations for contemporary, rapidly evolving PCa treatment modalities presents several challenges. These complexities arise from the wide range of available treatment options, which may be delivered in different settings (e.g., inpatient vs. outpatient, academic vs. non-academic institutions, etc.) and across varying delivery sessions (e.g., single vs. multiple). The heterogeneity of the localized PCa patient population, such as differences in cancer characteristics, comorbidities, differences in patient values and decision-making process, and racial disparities, further complicates such analyses [23,24,25,26]. Additional factors influencing outcomes include the choice of time horizon (short- vs. long-term), the selection of cost components (e.g., diagnostics, consultations, re-admissions, lost workdays), and whether the focus is on oncological or functional outcomes [27]. These challenges are compounded by the broader complexities inherent to economic evaluations, such as determining the appropriate analytical perspective (e.g., payer, healthcare system, societal) and selecting the most suitable framework, whether cost-comparison, cost-effectiveness, or cost-benefit analysis [28,29].

Our results are limited by the following factors. First, we did not include post-treatment costs, as some patients followed up with their local physicians, resulting in missing data on post-operative emergency room visits, re-admissions, complication management, and possible adjuvant therapy. Second, the study period was short; however, this was to avoid confounding effects from the COVID-19 pandemic. Third, while the study adopted a healthcare system perspective, we could not account for out-of-pocket costs due to the heterogeneity of data and variability of insurance coverage across patients and treatment modalities.

## 5. Conclusions

Significant variation is observed in associated costs across treatment modalities for localized PCa, with the highest cost seen in IMRT and SBRT, followed by RALP, HIFU, LDBT, Cryo, and RRP. The number of sessions and technological complexity are significantly correlated with increased costs, highlighting the need to weigh economic considerations in treatment planning. Studies that evaluate costs alongside functional and oncological outcomes across multiple modalities are necessary to draw more definitive conclusions.

## Figures and Tables

**Table 1:** Captured costs associated with each treatment modality.

IMRT, SBRT	Fiducial Placement ± Spacer Gel Injection, Simulation, Dosimetry, IMRT/SBRT Delivery Until Last Session
LDBT	Prostate volume study, Simulation, BT seed Implanting, Dosimetry to discharge.
RALP, RRP	Surgical procedure to discharge.
HIFU *, Cryo *	Ablation procedure to discharge.

* We also captured the costs of post-ablation surveillance MRI and possible biopsy, which are not included in the comparison and are reported separately. Abbreviations: Cryo: Cryotherapy; HIFU: High-Intensity Focused Ultrasound; IMRT: Intensity-Modulated Radiation Therapy; LDBT: Low-Dose Rate Brachytherapy; SBRT: Stereotactic Body Radiation Therapy.

**Table 2:** Baseline age, race, and Gleason grade groups of included patients. The number of sessions for each modality is also reported.

Modality N (%)		IMRT 65 (12)	SBRT 17 (3)	RALP 212 (38)	HIFU 28 (5)	LDBT 42 (8)	Cryo 40 (7)	RRP 146 (26)
Age (Mean ± SD)		69 ± 7	67 ± 6	63 ± 7 ^†^	66 ± 8	65 ± 5 ^†^	69 ± 6	63 ± 7 ^†^
Race n (%)	White	36 (55) ^ƒ^	13 (76)	140 (66)	20 (71)	27 (64)	21 (53)	115 (79) ^ƒ^
	Black	26 (40)	3 (18)	56 (26)	5 (18)	15 (36)	17 (42)	27 (19)
	Others/not disclosed	3 (5)	1 (6)	16 (8)	3 (1)	0 (0)	2 (5)	4 (2)
NCCN Risk Group n (%)	Low	4 (6) ^‡^	0 (0) ^‡, µ, ££^	39 (19) ^µ^	3 (11)	7 (17) ^‡^	1 (2) ^£^	31 (21) ^£, ££^
	Favorable- Intermediate	18 (28)	14 (82)	79 (37)	16 (57)	24 (57)	24 (60)	42 (29)
	Unfavorable- Intermediate	43 (66)	3 (18)	94 (44)	9 (32)	11 (26)	15 (38)	73 (50)
Number of Sessions *		20, 28	5	1	1	3–4	1	1

^†^ Denotes statistically significant difference from IMRT and Cryo; *p* < 0.0024 was considered statistically significant to account for the Bonferroni correction of 21 pairwise Mann–Whitney comparisons (0.05/21 = 0.0024). ^ƒ^ Indicates the only statistically significant difference (*p* < 0.0024) in race distributions was detected between the RRP group vs. IMRT. ^‡^ Denotes the statistically significant difference (*p* < 0.0024) between IMRT vs. RALP and LDBT cohorts, ^µ^, ^£^ and ^££^ indicate the same between the RALP vs. SBRT groups, Cryo vs. RRP groups, and RRP vs. SBRT cohorts, respectively. * IMRT and SBRT required 2–3 sessions for fiducial placement, simulation, and dosimetry. In some cases, the first two procedures are delivered on the same day. Abbreviations: Cryo: Cryotherapy; HIFU: High-Intensity Focused Ultrasound; IMRT: Intensity-Modulated Radiation Therapy; LDBT: Low-Dose Rate Brachytherapy; RALP: Robotic-Assisted Laparoscopic Prostatectomy; RRP: Radical Retropubic Prostatectomy; SBRT: Stereotactic Body Radiation Therapy.

**Table 3:** Mean total costs, direct costs, and total charges of PCa treatment for each modality.

Modality	Cases N (%)	Cases Total Costs (USD) Mean ± SD	Direct Costs (USD) Mean ± SD	Total Charges (USD) Mean ± SD
IMRT	65 (12)	28,487 ± 5970	17,703 ± 5501	136,201 ± 10,061
SBRT	17 (3)	20,266 ± 1701	10,585 ± 1103 ^ƒ^	121,254 ± 6927
RALP	212 (38)	14,021 ± 3333 ^†^	10,135 ± 1996 ^ƒ^	40,331 ± 6628 ^µ^
HIFU	28 (5)	12,895 ± 1545 ^†, ‡^	8919 ± 997 ^£^	40,590 ± 3733 ^µ^
LDBT	42 (8)	12,682 ± 1685 ^‡^	8065 ± 1106 ^£^	65,518 ± 2778
Cryo	40 (7)	10,456 ± 1399	7087 ± 997	33,166 ± 4318
RRP	146 (26)	9749 ± 2561	6148 ± 1653	25,562 ± 6406

Currency is in U.S. dollars. In the costs and charges columns, all values in each column are statistically significantly different (*p* < 0.0024) from other rows based on the Mann–Whitney tests, except those marked with the same superscripts (^†, ‡, £, ƒ, µ^). Abbreviations: Cryo: Cryotherapy; HIFU: High-Intensity Focused Ultrasound; IMRT: Intensity-Modulated Radiation Therapy; LDBT: Low-Dose Rate Brachytherapy; RALP: Robotic-Assisted Laparoscopic Prostatectomy; RRP: Radical Retropubic Prostatectomy; SBRT: Stereotactic Body Radiation Therapy.

## Data Availability

The de-identified data used for this study is available upon reasonable request from the corresponding author.

## Supplementary Materials

The following Supporting Information can be downloaded at: https://www.mdpi.com/article/10.3390/cancers17172814/s1, Table S1: The Duke Finance database was queried for “C61” ICD-10-CM codes. We then identified patients in surgical modality cohorts using CPT codes, which were subsequently verified through a manual review. For radiation modalities, we filtered by charge codes and confirmed eligibility using CPT codes and a hand search; Table S2: Direct cost, total cost, and total charges of brachytherapy implant placement, prostate volume study, simulation, and post-implant dosimetry separated; Table S3: (a) Direct cost, total cost, and total charges of IMRT delivery with and without spacer gel injection. These amounts were also separated for the 28 versus 20 sessions of IMRT delivery, (b) Breakdown of fixed direct, fixed indirect, variable direct, and variable indirect costs for the IMRT cohort with 28-session treatment based on the location of the fiducial placement session; Table S4: Breakdown of fixed direct, fixed indirect, variable direct, and variable indirect costs for the SBRT cohort with and without spacer gel injection based on the location of the fiducial placement session; Table S5: Mean total costs, direct costs, and total charges of PCa treatment for Cryo and HIFU stratified by focal versus whole-gland ablation; Table S6: Direct cost, total cost, and total charges for cases who underwent surveillance multiparametric MRI and/or biopsy, among Cryo and HIFU cohorts.

## Abbreviations

The following abbreviations are used in this manuscript:

PCa	Prostate Cancer
Cryo	Cryotherapy
HIFU	High-Intensity Focused Ultrasound
LDBT	Low-Dose Brachytherapy
RALP	Robotic-Assisted Radical Prostatectomy
EBRT	External-Beam Radiation Therapy
TULSA	MRI-Guided Transurethral Ultrasound Ablation
AS	Active Surveillance
RRP	Open Retropubic Radical Prostatectomy
IMRT	Intensity-Modulated Radiation Therapy
SBRT	Stereotactic Body Radiotherapy
ICD-10-CM	International Classification of Diseases, Tenth Revision, Clinical Modification
CPT	Current Procedural Terminology
NCCN	National Comprehensive Cancer Network
SD	Standard Deviation
MRI	Multiparametric MRI
SPSS	Statistical Package for the Social Sciences
RP	Radical Prostatectomy
