# Supplementary material for: Comparative Economic Evaluation of Radical Prostatectomy, Radiation, and Ablative Techniques in the Management of Localized Prostate Cancer
Source: Cancers (Basel). 2025 Aug 28;17(17):2814. doi: 10.3390/cancers17172814 (PMC12427179; doi:10.3390/cancers17172814)
Supplement: Supplementary file 1 [file cancers-17-02814-s001.zip › cancers-3818428-supplementary.pdf]

Supplementary Tables

Table S1. The Duke Finance database was queried for "C61" ICD-10-CM codes. We then identified patients in surgical modality cohorts using CPT codes, which were subsequently verified through a manual review. For radiation modalities, we filtered by charge codes and confirmed eligibility using CPT codes and a hand search.

	IMRT	SBRT	RALP	HIFU	LDBT	Cryo	RRP
CPT Codes	--	--	55866	C9747	--	55873<br>C2618	55840<br>55842<br>55845
Charge Codes*	✓	✓	--	--	✓	--	--
manual Confirmation	✓	✓	✓	✓	✓	✓	✓

* Charge codes are not reported because they were institution-specific. Abbreviations: Cryo: Cryotherapy; HIFU: High-Intensity Focused Ultrasound; IMRT: Intensity-Modulated Radiation Therapy; LDBT: Low-Dose Rate Brachytherapy; RALP: Robotic-Assisted Laparoscopic Prostatectomy; RRP: Radical Retropubic Prostatectomy; SBRT: Stereotactic Body Radiation Therapy.

Table S2. Direct cost, total cost, and total charges of brachytherapy implant placement, prostate volume study, simulation, and post-implant dosimetry separated.

LDBT	Cases (*n*)	TOTAL COSTS ($)	DIRECT COSTS ($)	TOTAL CHARGES ($)
Seed Placement				
Mean ± SD	42	8447 ± 1149	5010 ± 764	48,928 ± 2545
Median (IQR)		8538 (1620)	4989 (1370)	48,310 (4067)
Prostate Volume Study				
Mean ± SD	42	2220 ± 766	1463 ± 645	11,309 ± 1243
Median (IQR)		2639 (1501)	1821 (1221)	10,143 (1269)
Simulation				
Mean ± SD	42	1454 ± 302	1227 ± 256	3179 ± 665
Median (IQR)		1500 (35)	1267 (36)	3291 (0)
Post-implant Dosimetry				
Mean ± SD	42	561 ± 120	366 ± 83	2102 ± 0.0
Median (IQR)		515 (0.0)	328 (0.0)	2102 (0.0)

Abbreviations: LDBT: Low-dose brachytherapy.

Table S3. a: Direct cost, total cost, and total charges of IMRT delivery with and without spacer gel injection. These amounts were also separated for the 28 versus 20 sessions of IMRT delivery. b: Breakdown of fixed direct, fixed indirect, variable direct, and variable indirect costs for the IMRT cohort with 28-session treatment based on the location of the fiducial placement session.

(a)

IMRT (Overall)	Cases (*n*))	TOTAL COSTS ($)	DIRECT COSTS ($)	TOTAL CHARGES ($)
With Spacer Injection				
Mean ± SD	14	24,022 ± 6300	13,283 ± 6084	142,167 ± 10,135
Median (IQR)		23,490 (7121)	15,601 (9096)	141,575 (16,014)
Without Spacer Injection				
Mean ± SD	51	29,712 ± 5309	18,916 ± 4705	134,565 ± 9495

Median (IQR)		29,512 (6917)	18,048 ± 7075	131,327 (17,064)
p-value (Mann-Whitney)		< 0.001	< 0.001	0.062

Subgroups based on treatment sessions				
IMRT (Divided)	Cases (*n*))	TOTAL COSTS ($)	DIRECT COSTS ($)	TOTAL CHARGES ($)
28 Session (With Spacer Injection)				
Mean ± SD	10	25,158 ± 7204	16,095 ± 4719	146,722 ± 8035
Median (IQR)		26,708 (10,282)	16,124 (2073)	142,449 (16,435)
28 Sessions (Without Spacer Injection)				
Mean ± SD	49	30,097 ± 5052	19,404 ± 4104	135,459 ± 8444
Median (IQR)		29,905 (5052)	18,072 (6639)	131,328 (17,276)
p-value (Mann-Whitney)		0.008	0.011	0.004
20 Session (With Spacer Injection)				
Mean ± SD	4	21,181 ± 1121	6254 ± 1180	130,777 ± 3047
Median (IQR)		20,656 (1742)	5737 (1906)	130,854 (5898)
20 Sessions (Without Spacer Injection)				
Mean ± SD	2	20,300 ± 122	6964 ± 1091	112,654 ± 9261
Median (IQR)		20,300 (NA)	6964 (NA)	112,654 (NA)
p-value (Mann-Whitney)		0.121	0.245	0.053

(b)

**IMRT (Overall)**	**Cases (*n*))**	**Location**	**TOTAL COSTS ($)**	**DIRECT COSTS ($)**	**TOTAL CHARGES ($)**
With Spacer					
Mean ± SD	10	DRH*	25,158 ± 7204 †	16,095 ± 4719 †	146,722 ± 8035 †
Median (IQR)			26,708 (10,282)	16,124 (2073)	142,449 (16,435)
Without Spacer					
Mean ± SD	30	DCC**	28,018 ± 4135 †	17,522 ± 2416 †	138,246 ± 8461 †
Median (IQR)			28,192 (3400)	17,182 (1930)	132,364 (16,722)
Mean ± SD	19	ASC-OR**	33,378 ± 4685	22,376 ± 4502	131,058 ± 6436
Median (IQR)			34,129 (1117)	23,555 (1029)	128,580 (5077)
p-value (Mann-Whitney)			0.036 (DRH vs. DCC)	0.042 (DRH vs. DCC)	0.054 (DRH vs. DCC)
			0.004 (DRH vs. ASC-OR)	0.006 (DRH vs. ASC-OR)	< 0.001 (DRH vs. ASC-OR)
			< 0.001 (DCC vs. ASC-OR)	< 0.001 (DCC vs. ASC-OR)	< 0.001 (DCC vs. ASC-OR)

*Done by the interventional radiology team. **Done by the urology team. In the costs and charges columns, all mean values in each column are statistically significantly different ($p < 0.017$) from other rows based on the Mann-Whitney tests, except those marked with the same superscripts (†). p<0.017 was considered statistically significant to account for the Bonferroni correction of three pairwise Mann-Whitney comparisons (0.05/3=0.017). Abbreviations: ASC-OR: Duke Ambulatory Surgery Center Operative Room; DCC: Duke Cancer Clinic; DRH: Duke Regional Hospital; IMRT: Intensity-Modulated Radiation Therapy. NA: Not applicable.

Table S4. Breakdown of fixed direct, fixed indirect, variable direct, and variable indirect costs for the SBRT cohort with and without spacer gel injection based on the location of the fiducial placement session.

**SBRT (Overall)**	**Cases (*n*))**	**Location**	**TOTAL COSTS ($)**	**DIRECT COSTS ($)**	**TOTAL CHARGES ($)**
With Spacer					
Mean ± SD	8	DRH*	18,864 ± 341	9763 ± 240	127,922 ± 3586
Median (IQR)			18,729 (96)	9667 (81)	126,600 (430)
Without Spacer					
Mean ± SD	9	DCC**	21,512 ± 1405	11,315 ± 1052	115,327 ± 858
Median (IQR)			20,997 (2622)	10,870 (1814)	115,782 (1078)
p-value (Mann-Whitney)			0.001	0.001	0.001

* Done by the interventional radiology team. **Done by the urology team. Abbreviations: ASC-OR: Duke Ambulatory Surgery Center Operative Room; DCC: Duke Cancer Clinic; DRH: Duke Regional Hospital; SBRT: Stereotactic Body Radiation Therapy.

Table S5. Mean total costs, direct costs, and total charges of PCa treatment for Cryo and HIFU stratified by focal versus whole-gland ablation.

**Modality**	**Ablation Pattern**	**Cases *n*) (%)**	**Cases Total costs ($) Mean ± SD**	**DIRECT COSTS ($) Mean ± SD**	**TOTAL CHARGES ($) Mean ± SD**
Cryo*	Focal	12	10,551 ± 1822	7201 ± 1337	34,200 ± 5470
	Whole-Gland	28	10,415 ± 1212	7037 ± 836	32,723 ± 3751
HIFU*	Focal	19	12,490 ± 1207	8619 ± 753	39,842 ± 1967
	Whole-Gland	9	13,752 ± 1887	9554 ± 1189	42,172 ± 5845

* The Mann-Whitney test did not show a significant difference between the focal and whole-gland subgroups for each modality. Abbreviations: Cryo: Cryotherapy; HIFU: High-Intensity Focused Ultrasound.

Table S6. Direct cost, total cost, and total charges for cases who underwent surveillance multiparametric MRI and/or biopsy, among Cryo and HIFU cohorts.

	**Cases (*n*))**	**TOTAL COSTS ($)**	**DIRECT COSTS ($)**	**TOTAL CHARGES ($)**
Post-Ablation mpMRI				
Mean ± SD	38	888 ± 190	458 ± 108	6473 ± 1722
Median (IQR)		894 (171)	474 (112)	6695 (495)
Post-ablation Biopsy				
Mean ± SD	17	3967 ± 970	2550 ± 546	9001 ± 4459
Median (IQR)		3819 (337)	2471 (382)	7285 (1774)
